# Associates of Respite Care Utilization Among Family Caregivers of Older Adults in the US

**DOI:** 10.1093/geroni/igaf122.850

**Published:** 2025-12-31

**Authors:** Jun Chu, Joseph Svec, Fei Sun

**Affiliations:** University of Maryland, Baltimore County, Baltimore, Maryland, United States; St. Joseph’s University, Patchogue, New York, United States; Michigan State University, East Lansing, Michigan, United States

## Abstract

Respite care provides a relief to family caregivers by temporarily stepping in to provide care for older adults. Prior literature has shown that respite care services can improve both physical and mental health outcomes for caregivers and care recipients. Despite these benefits, respite utilization remains low, and little is known about the profiles of caregivers and care recipients who uses respite care. Using 2015, 2017 and 2021 National Health and Aging Trends Study (NHATS) and National Study of Caregiving (NSOC), we examined the prevalence and associates of respite usage among family caregivers of older adults. Our study sample represented weighted 15,329,939 caregivers, with average age around 59.14 years. 16.45% caregivers used respite care services. Findings revealed that caregivers with higher income and education levels were more likely to use respite. Those with higher emotional burdens, including feeling little joy in caregiving (6.0 percentage points more, p < 0.001), and struggling emotionally with caregiving responsibilities (5.62 percentage points more, p < 0.001), were more likely to use respite. Older care recipients with Medicaid coverage (5.8 percentage point more, p < 0.01), dementia diagnosis (9.76 percentage points more, p < 0.001) and more chronic conditions (5.23 percentage points more, p = 0.02) were positively associated with respite usage of caregivers. Our results suggests the current respite care demand is driven by caregivers’ desire to reduce caregiving burden, as well as the care recipient’s Medicaid status. The results underscore the need for expanded respite care funding and policies to improve access for low-income caregivers and those with emotionally and financially challenging circumstances.

